# High-throughput genotyping of high-homology mutant mouse strains by next-generation sequencing

**DOI:** 10.1016/j.ymeth.2020.10.011

**Published:** 2021-07

**Authors:** Diane Gleeson, Debarati Sethi, Radka Platte, Jonathan Burvill, Daniel Barrett, Shaheen Akhtar, Michaela Bruntraeger, Joanna Bottomley, Sanger Mouse Genetics Project, James Bussell, Edward Ryder

**Affiliations:** Wellcome Sanger Institute, Wellcome Genome Campus, Hinxton, Cambridge CB10 1SA, UK

**Keywords:** CRISPR, clustered regularly interspaced short palindromic repeat, het, heterozygous, hom, homozygous, NGS, next generation sequencing, NHEJ, non-homologous end joining, PCR, polymerase chain reaction, QC, quality control, sgRNA, single guide RNA, WT, wild-type, Genotyping, Mouse, NGS, CRISPR, Mutant, QC

## Abstract

Genotyping of knockout alleles in mice is commonly performed by end-point PCR or gene-specific/universal cassette qPCR. Both have advantages and limitations in terms of assay design and interpretation of results. As an alternative method for high-throughput genotyping, we investigated next generation sequencing (NGS) of PCR amplicons, with a focus on CRISPR-mediated exon deletions where antibiotic selection markers are not present. By multiplexing the wild type and mutant-specific PCR reactions, the genotype can be called by the relative sequence counts of each product. The system is highly scalable and can be applied to a variety of different allele types, including those produced by the International Mouse Phenotyping Consortium and associated projects.

One potential challenge with any assay design is locating unique areas of the genome, especially when working with gene families or regions of high homology. These can result in misleading or ambiguous genotypes for either qPCR or end-point assays. Here, we show that genotyping by NGS can negate these issues by simple, automated filtering of undesired sequences. Analysis and genotype calls can also be fully automated, using FASTQ or FASTA input files and an in-house Perl script and SQL database.

## Highlights

• Next generation sequencing is a scalable solution to genotyping mutant mice.
• Ratios of wild type and mutant sequence counts are used to call the genotype.
• Hundreds of samples can be multiplexed into one sequencing experiment.
• Amplification of high-homology genes can be easily filtered out during analysis.

## 1 Introduction

The mouse remains a vitally important model in studying gene function [1] and modelling human disease [2]. A firm commitment to the 3Rs [3] and ARRIVE guidelines [4] from mouse production centers to ensure validity and reproducibility in experiments [5], dictate that over-production of breeding cohorts or repeats of phenotyping should be avoided whenever possible. Even after standardization of variables such as genetic background, husbandry regimes, and data analysis [6], possible sources of error include misidentification of mice, incorrectly targeted or poorly characterized mutations, and incorrect genotyping strategies or assay design. The longer these issues take to resolve, the more costly they are in terms of animal use, lost research time and housing. Similarly, delays in genotyping turnaround time can have downstream effects on breeding decisions and cage use. Accurate, timely, and cost-effective genotyping of mice is therefore of utmost importance to facilitate correct cohort breeding and phenotyping analysis.

### 1.1 Large scale mouse production initiatives.

In the last ten years there has been a move away from individual laboratories creating knockout mouse models to a more centralized approach, highlighted by the Sanger Institute Mouse Genetics Project (MGP) [7], EUCOMM/KOMP [8], EUMODIC [9], and the International Mouse Phenotyping Consortium (IMPC) [10]. These projects have been highly successful, forming the basis of high-throughput screens studying developmental phenotypes [11], cancer metastasis [12], sexual dimorphism [13], hearing loss [14], metabolism [15], placental defects [16], and transcriptome signatures during development [17]. The mouse strains generated are freely available to the research community through repositories such as Infrafrontier [18] and MMRRC [19].

The advantages of this approach are cost-saving at scale, reduced numbers of mice used due to standardized and optimal breeding, and a unified approach to mouse quality control. As the IMPC moves into its third phase of research, focusing on precision models [20] and biological function of all genes [21], accurate quality control and genotyping of possibly complex mutation outcomes [22], [23], [24] will be paramount.

### 1.2 Allele QC and genotyping techniques

**Fig. 1:**
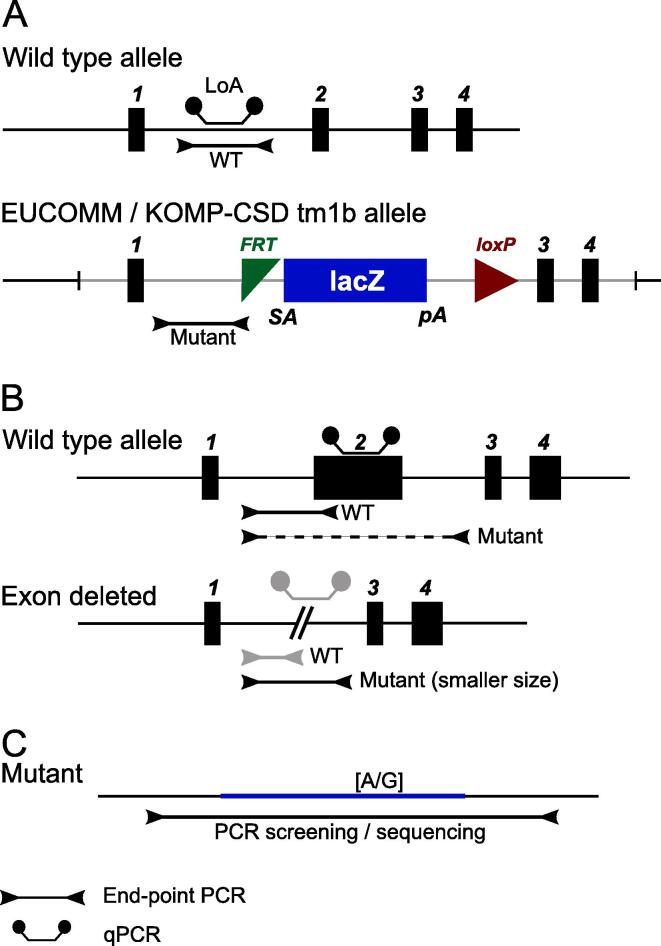
Common knockout designs. End-point PCR assays for EUCOMM/KOMP-CSD mutants are designed to amplify either the wild type or mutant alleles and fail if the allele is not present. Exon deletions follow this same premise, although the mutant assay may amplify a larger size if the exon is sufficiently small. For SNP detection, a single assay spans the region of interest.

The mouse production initiatives discussed above historically used the LacZ gene-trapping tm1a cassette and derivative alleles created by EUCOMM/KOMP-CSD [25], with a switch to CRISPR/Cas9 mutations [26] (primarily exon deletions) as the technology became available and optimized for large-scale mutagenesis [27]. Whereas tm1a alleles could be genotyped in a high-throughput manner using a qPCR assay designed to the LacZ or Neo markers within the cassette [7], exon deletions carry no such signatures (Fig. 1). Genotyping deletions therefore relies predominantly on either end point PCR [28] or loss of wild type allele qPCR (LoA) [29], [30] which uses an assay designed to the wild type locus and compares the amplification of an unknown sample against a calibration control.

Alternative forms of routine genotyping include Kompetitive allele specific PCR (KASP) assays [31], High Resolution Melt Analysis (HRMA) [32] and fluorescent PCR-capillary gel electrophoresis [33]. Although KASP assays have been used to genotype SNPs and small indels from NHEJ CRISPR experiments [34], their use in exon deletions has not yet been explored.

Quality control of mouse mutant strains often takes a multi-assay and multi-technique approach, which is vital for situations where results may be confusing or conflicting [35]. Southern blot has been accepted as the gold standard of characterizing mutations based on homologous recombination [36] but is time consuming and difficult to adapt to a high-throughput environment. Alternatively, digital droplet PCR (ddPCR) has been used as an alternative to pre-microinjection QC for karyotyping [37] and evaluation of CRISPR mutants [38]. Although excellent for evaluation of a mutation, limitations in throughput and cost per sample makes digital droplet PCR less desirable for routine genotyping.

The clonal amplification technique used in NGS makes it a valuable tool for assessing the outcome of small, indel-based knockout CRISPR/Cas9 experiments [39], where compound alleles can make interpretation of capillary sequencing, and thus which mice to breed, very challenging in the G0 generation. Third-generation, long-read sequencing has also been used to determine allele structure in gene editing outcomes of more complex alleles [24].

### 1.3 Quality control and confirmation of homozygotes

One potential drawback of end point PCR and LoA qPCR genotyping, where an assay is designed to fail in the presence of the wild type allele, is that non-specific amplification may give a false result. Thus, strains which are actually homozygous viable may appear as homozygous lethal when genotyped. Since allele QC is performed initially on heterozygous founders, these problems may not be detected until non-Mendelian ratios of genotypes start appearing in heterozygote cohort breeding. This issue is particularly acute in gene families, where high homology to other regions of the genome can severely limit assay design.

### 1.4 Genotyping challenges in regions of high homology and gene families

Analysis of mouse gene paralogues from Ensembl v101 data [40] (not including olfactory receptors, vomeronasal genes, or genes on the Y chromosome) revealed that 2829 genes have over 80% identity to one or more genes within the genome (12.5% total). Raising the threshold to 90% identity reduces this to 1942 genes, but still represents approximately 9.5% of the genome. This not only has implications for selecting unique guide RNA design in creating mutant strains, but also designing specific genotyping assays. Two examples of mouse strains produced by the Sanger Institute Mouse Pipelines teams are derived from the Psg and Sirpb1 gene families.

The mouse pregnancy-specific glycoprotein (Psg) family consists of 17 members [41]. The mouse locus is approximately 1.74 Mb and contains six Psg genes in the A2 chromosome band and eleven genes in the A3 band on Chr 7 [42]. The signal-regulatory protein beta Sirpb1a-c family resides on a 0.5 Mb portion of the A1 region on Chr 3. Genes in this family have over 90% sequence homology to each other and 48% homology with the nearby gene Gm5150 [40].

### 1.5 Genotyping by next generation sequencing

In addition to mouse whole-genome [43] and exome sequencing [44], massively parallel amplicon sequencing has been used to classify the mouse gut microbiome [45] and gene editing outcomes [46]. Although the concept of using the extensive capacity of NGS to sequence single PCR products for mouse genotyping seems counter-intuitive, careful multiplexing of libraries can provide hundreds of samples in one sequencing run, vastly reducing the sequencing cost per sample. Coupled with the additional information sequencing provides, decreasing costs of reagents and cheaper, smaller capacity instruments to reduce potential batching times, NGS becomes a more feasible proposition.

To investigate the utility of Illumina-based sequencing for mouse genotyping, especially in challenging regions of high homology and gene families, we performed a pilot study on twenty-six mutant strains and compared the results to our existing qPCR assays.

## 2 Materials and methods

The care and use of all mice in this study were in accordance with the UK Home Office regulations, UK Animals (Scientific Procedures) Act of 1986, and were approved by the Wellcome Trust Sanger Institute Ethical Review Committee.

### 2.1 Gene choice

Genes for this study were chosen based on sample availability, existing genotyping issues, or homology to other genes. In addition to the CRISPR/Cas9 exon deletions, we chose one CRISPR/Cas9-derived SNP and two EUCOMM/KOMP tm1b strains [47] to test the versatility of the system (Table 1).

**Table 1:** Genes and allele types used in the study which were successfully amplified in the NGS pipeline. Genes highlighted in bold are characterized as high-homology (greater than 90% homology over the assay design area).

Mutation type	Genes
EUCOMM/KOMP tm1b	Afap1l1, Id2
SNP	Pcdh15
Exon deletion	Ceacam15, Clic3, Crip1, Etv3, Exo5, Gm17750, Itgam, Msl1, **Pilrb2**, **Psg18**, **Psg19**, **Psg21**, **Psg26**, **Sirpb1a**, **Sirpb1b**, **Sirpb1c**, Sqrdl, Stom, **Vmn2r27**, Zfp748

### 2.2 Assay design

The rationale of assay designs for the different mutations are outlined in Fig. 1. A combination of end point and LoA qPCR is used to call and confirm the genotype. Primer sequences are shown in Supplemental Table ST1.

### 2.3 DNA extraction

DNA was isolated from ear punches taken as part of the mouse identification process, using the TaqMan® Sample-to-SNP^TM^ kit (Thermo Scientific). Ear clips were heated to 95 °C for 3 min in 50 µl of lysis buffer, centrifuged briefly and neutralized with the addition of 50 µl of stabilizing solution. Extracted DNA was stored at −20 °C until required.

### 2.4 Two-round PCR

**Fig. 2:**
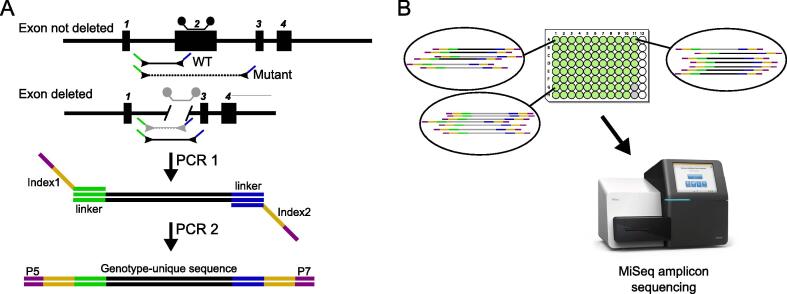
NGS genotyping strategy for CRISPR-derived exon deletions. A) In PCR 1, tailed primers containing a linker sequence are amplified in a WT and mutant-specific multiplex reaction. A second round of PCR is then performed using additional tailed primers containing flow cell adaptors and an 8 bp barcode index sequence at each end. B) By using plate-specific and well-specific barcodes, many plates can be multiplexed into one sequencing run. After the sequencing by synthesis is complete, the MiSeq then deconvolutes the clusters and assigns the results to FASTQ files per sample, based on the barcode sequence.

Amplicon-derived sequencing libraries were constructed using a 2-step PCR method (Fig. 2), similar to that used in metagenomics studies [48] and determining CRISPR/Cas9 editing outcomes [49], [50]. Gene-specific primers (Table ST1) were tagged at the 5′ end with the following sequences: PE_forward primer _F1 ACACTCTTTCCCTACACGACGCTCTTCCGATCT, PE_reverse primer_R1 CGGTCTCGGCATTCCTGCTGAACCGCTCTTCCGATCT. Round 1 amplification was performed in a multiplex reaction of all three genotyping primers using Platinum Taq (Thermo Scientific) and an annealing temperature of 58 °C (Supplemental Data S1). Where possible, existing primers from our mutation QC steps were used as the basis of the PCR reaction. If the forward primer was greater than 150 bp from the discriminatory sequence motif, or the amplicon larger than 250 bp, then new primers were designed. Reactions were visualized using a Qiaxcel Advanced capillary electrophoresis instrument (Qiagen) and DNA Screening Kit, and genotypes called on the amplification pattern of wild type and mutant alleles.

The second round of PCR was performed as described in Bruntraeger et al. [49]. Briefly, reactions from round 1 are amplified in the presence of a high-fidelity polymerase and primers with P5 or P7 flow cell attachment sequences, 8 bp barcodes, and sequences identical to the round 1 PCR tag. Each 96 well plate contains a constant i5 index and a unique i7 index which allows for many samples and plates to be multiplexed on one MiSeq run, increasing throughput and decreasing costs per sample per run. Aliquots of each second round reaction were pooled and purified by use of Ampure XP bead (Beckman Coulter) size selection [51]. Sequencing was performed by the Sanger Institute DNA Pipelines teams, using a MiSeq instrument (Illumina) and a MiSeq Reagent Kit v2 (300-cycles) (Illumina).

### 2.5 Real time qPCR

FAM-labelled TaqMan™ assays were designed using Primer Express 3 (Thermo Scientific) and obtained from Thermo Scientific. Reactions were performed on a Viia7 instrument using GTXpress master mix in the presence of an endogenous control VIC-labelled Tfrc assay (Thermo Scientific). Reaction and cycling conditions are shown in Supplemental Data S2. Genotypes were called using the 2^−ΔΔCt^ method [52], comparing against known heterozygous and wild type calibration controls.

### 2.6 Sequence analysis

**Fig. 3:**
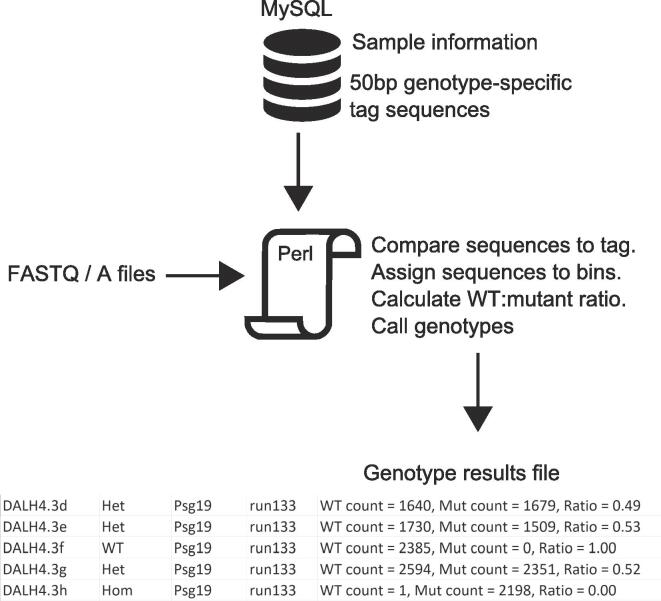
Data analysis pipeline. Mouse ID and strain information is retrieved from the MySQL database by processing the FASTQ filename. Sequences are compared to a database of genotype-specific motifs, any matches reported, and the genotype called on the ratio. FASTQ files can be pre-filtered on the quality score if required.

Sequence data analysis and genotype calling is performed by a custom Perl script, which accepts directories of sequence files in either FASTQ or FASTA format (Fig. 3). The script connects to a MySQL database containing mouse and gene ID, and compares each sequence to a wild type and mutant-specific 50 bp motif (Supplemental Table ST2). By this stage in the colony expansion the mutations and breakpoints have been characterized, so re-alignment of sequences to the genome using software such as Crispresso2 [53] is not necessary, and may prove problematic due to the deletion size. A motif-based approach therefore offers a simpler method of analysis and potential increases in computational speed.

Matches are assigned to bins, the ratio of wild type to mutant sequences calculated, and the genotype reported in a tab-delimited file. Genotypes are set to failed if the maximum number of sequence reads in both bins were below 500. In the case of CRISPR exon deletions where the mutant-specific assay is amplified in the wild type allele (albeit a different size), a filter motif removes the sequence from the analysis. The analysis presented here was performed primarily using the R1 sequence, but the Perl script can also process the R2 end if required. The Perl scripts and database schema are available from GitHub at (https://github.com/EdRyder/ngs-genotyping).

## 3 Results.

### 3.1 PCR amplification

In total, 23/26 genes tested (88%) amplified a product without any additional reaction optimization beyond our existing workflows (Table 1). Between twelve and twenty-four samples of each mouse strain were genotyped, chosen on the basis of a range of existing genotypes and litter sizes to facilitate sample processing. An example of results for the CRISPR-derived Ceacam15 exon deletion strain is shown in Table 2.

**Table 2:** Ceacam15 genotyping, comparing the Qiaxcel end-point PCR genotypes, LoA qPCR and NGS results. NGS ratios are consistent with previous LoA qPCR genotype calls. Mice are identified by colony prefix, mating, litter and individual. The LoA score is reported here as the relative quantification (RQ) value, where 1 = amplification identical to WT controls and 0 = no amplification (homozygote). Heterozygotes are called where the RQ value is in a defined range of 0.5. PCR gel sizes: WT = 141 bp, mutant = 162 bp.

To demonstrate the high-throughput potential of next-generation sequencing for this application, one of the experiments included the pooling of 768 mice from 25 strains within one sequencing run.

### 3.2 NGS genotyping ratios

**Fig. 4:**
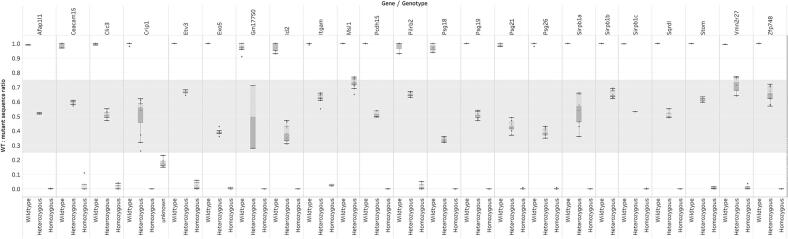
Genotyping results per mouse strain using next generation sequencing. Ratios are calculated by comparing the number of wild type motif sequence hits against mutant motif hits. The shaded area represents the ideal range for heterozygous genotypes.

A graph of sequence ratios per gene is shown in Fig. 4. Three distinct clusters were formed in most cases, and average WT:mutant ratios for heterozygotes ranged from 0.39 to 0.729 (Supplemental Data S3), reflecting possible allele-specific bias in amplification of the two products. For the NGS assays, the average difference between WT and mutant assays was 16 bp, with a median of 10 bp (assays were originally designed to preserve a minimum size difference). No clear pattern was observed between the difference in WT and mutant amplicon sizes, and the heterozygote genotype ratio. The cause of the altered ratios may, therefore, be due to other factors such as secondary structure or GC content.

### 3.3 Comparisons with LoA qPCR results

Genotypes were compared with previous LoA qPCR results, splitting the genes into two groups based on whether they were highly homologous (>90%) to other genes over the design area (Table 3).

**Table 3:** Genotype comparisons between LoA qPCR and NGS sequence ratios. High homology genes have a lower genotype concordance than those with no homology, suggesting an issue with the specificity of the design in one or either assay.

	High homology gene	
NGS and LoA qPCR agree?	No	Yes
Yes	90%	82%
No	10%	18%

For the ‘no high homology’ genes, agreement between the two methods was 90% (231/262), which was lower than expected. Upon further investigation, the majority of failed matches belonged to the genes Gm17750 and Crip1 (8 and 13 mismatches, respectively). Amplification of Gm17750 suppressed the mutant assay in the NGS PCR compared to the wild type (data not shown), lowering the ratio scores and causing heterozygotes to appear as wild types in the analysis.

Interestingly, Crip1 formed a fourth distinct cluster of 5 mice with WT:Mut count ratios between 0.15 and 0.23. The reason for this is unclear, but all the affected mice were from the same litter (Crip1_7.2) and genotyped as homozygous by LoA qPCR (due to the band sizes of 185 bp and 186 bp for the wild type and mutant assays, the genotype could not be called by observing the end point PCR in this study). A separate litter of 11 mice (Crip1_15.3) all genotyped as expected, and contained a mix of homozygotes, heterozygotes, and wild type mice. As all mice in the breeding cohorts were derived from a single exon-deleted heterozygous founder (a characterized G1 male), this is unlikely to be the result of multiple mutations within the colony and may be due to a poor DNA preparation in the Crip1_7.2 litter. Removing Crip1 and Gm17750 from the analysis increased the percentage match with the LoA qPCR results to 98% in ‘no homology’ genes where a result was obtained for both samples.

### 3.4 Genotyping and filtering of high-homology targets

The concordance between NGS and LoA qPCR dropped to 82% when analyzing ‘high-homology’ genes, indicating potential issues with our existing qPCR-based method. LoA qPCR assays were originally designed to minimize the amount of homology with other genomic regions (which can be iterative and labor-intensive), while staying within the parameters required for successful TaqMan-based amplification and PCR efficiency.

LoA qPCR Assays with greater than 10 base mismatches to other regions (e.g. exon deletion strains Psg18, Psg19, Psg26) agreed with the NGS results. The Pilbr2 mutants also showed no issues at all on the mice tested, with 100% agreement between the LoA qPCR, and NGS genotyping, despite only 5 base mismatches across the 58 bp LoA assay.

We found a greater likelihood of the LoA qPCR and NGS assays producing conflicting results when the number of mismatches within the LoA qPCR assay were below 5 bp. Exon deletion strains for Psg21, Sirbp1a, Sirbp1c, and Vmn2r27 all showed serious issues in genotyping concordance, although Sirpb1b did not, despite an only 2 bp difference in the assay sequence.

The advantage of the NGS approach over LoA qPCR is the precise sequence of the products can be determined, allowing the filtering out of unintended amplification. Exon deletion Psg21 has a 97% sequence match to Psg23 across the entire 2.4 kb deletion and flanking areas and numerous matches to other Psg genes (e.g. Psg27) throughout the region (Supplemental Data S4). This makes designing specific qPCR assays extremely challenging, and in the case of Psg21 resulted in an assay differing in only 2 bp from Psg23 and Psg 27, albeit at different locations. While the end point, gel-based PCR results for Psg21 agreed with the LoA qPCR results, three mice from litter Psg21_3.2 (het × het mating) were called as clear homozygotes by our NGS pipeline (Table 4). To investigate further, we mapped the FASTQ file for Psg21_3.2a to the mouse genome (release mm10) using Bowtie2 [54]. From a total of 3702 mapped sequence reads corresponding to the wild type assay (the mutant-specific assay reported as unmapped due to the mutation), 2406 (65%) mapped to a homologous region in Psg23, and 1268 (34%) to Psg27. Mice from litter Psg21_2.1 (het × WT mating) all agreed with the existing genotypes.

**Table 4:** Psg21 genotyping results comparison. Two mice were genotyped as heterozygotes by LoA qPCR and from the PCR 1 reaction used in the NGS experiment. Further analysis, however, revealed that the WT band was spurious, and the true genotype was homozygous (shaded regions). PCR gel sizes: WT = 191 bp, mutant = 157 bp.

Three genes from the Sirpb1 family were also processed as part of the study, and although LoA qPCR and NGS genotyping agreed for Sirpb1b, Sirpb1c failed to detect NGS homozygotes by LoA qPCR, and the LoA assay for Sirpb1a was highly variable between litters. The litter Sirpb1a_9.1, for example, was reported as all heterozygotes by LoA qPCR but genotyped as all homozygous by NGS. The FASTQ file for Sirpb1a_9.1a, a heterozygote by LoA qPCR, was aligned to the genome and showed only 8 alignments to the WT Sirpb1a allele (and 0 hits for the motif region), but 2517 hits to Sirpb1c and 371 to Gm5150. From this data, we conclude that the true genotypes for this litter are homozygotes and that the LoA qPCR assay is amplifying non-specific products at a signal high enough to produce a false result.

### 3.5 Cross-contamination of samples and the effects on NGS sequence ratios

One advantage of qPCR over gel-based end point PCR methods is that the analysis method is resilient to low levels of cross-contamination, which can occur at any stage throughout the process of ear clipping to PCR experiment. In negative controls, wild type (cassette qPCR), or homozygote (LoA qPCR) samples, any amplification in this type of event is usually associated with a much higher cycle threshold (Ct) value than typically seen for the assay. The comparative analysis reports these as low copy numbers which are then filtered out of the genotype calculation.

To investigate the robustness of the next-generation sequencing approach, we spiked wild type or homozygous mice with DNA from the corresponding opposite genotype at different levels. Results are shown in Supplemental Data S5. A 1:1 ratio of WT:hom DNA gave values indistinguishable from heterozygotes as expected, with a shift in sequence ratios observed with increasing levels of spiked DNA. Spiking ratios of 1:500 and above were out of the genotype calling window for heterozygote mice for all genes tested.

## 4 Discussion

We present here the results of a pilot project to evaluate next generation sequencing for both routine genotyping and additional quality control of mutant mouse strains. The workflow has been tested primarily on CRISPR/Cas9-derived exon deletions, but also on EUCOMM/KOMP alleles and a SNP mutation to assess the versatility of the workflow. Twenty-three of twenty-six (88%) genes tested amplified a product without any additional reaction optimization beyond our routine end point PCR pipeline, despite the addition of tails over 30 bp to the 5′ end of each primer. No changes were required to the DNA isolation protocol, and primer design required minimal changes to be within 150 bp of the mutation boundary/motif region. Performing sequencing on the Illumina MiSeq with a larger cycle kit would relax these restrictions, but these were not available during the study.

In addition to the sequence-level confirmation of the assay tested, another advantage of next generation sequencing over other approaches is its scalability. By using a dual combinational indexing approach, hundreds of samples can be batched into one sequencing run (which typically takes fewer than 24 hrs on a MiSeq, less if only a single end is sequenced), greatly reducing sequencing costs per sample. Combining the 16 standard Illumina Nextera XT i5 indexes with the i7 indexes described in Bruntraeger et al. [49], for example, provides barcodes for 1536 samples within one sequencing run.

For high-throughput mouse programs such as the IMPC, any increase in turn-around time due to batching would, therefore, be marginal and within the weaning time (where mice are transferred into new cages) if the identification clips are taken in a timely manner. Comparative costs of NGS and LoA qPCR will depend greatly on equipment available to the genotyping facility, and genotyping workflows. For example, the costs associated with the extra step required for the indexing round in NGS may be offset by not requiring the fluorescent hydrolysis probe needed for LoA qPCR. With pipetting automation, labor costs per sample can also be kept low for both methods, and any existing high-throughput setup for LoA qPCR or end-point PCR should be easily adaptable to the sequencing approach.

Our analysis script greatly simplifies the processing of results and can be used with minimal training. Additionally, the tab-delimited output format allows results to be easily processed downstream and uploaded into a mouse tracking database if required. Although the Bioperl module for FASTQ processing can take several minutes per file (depending on size), FASTA format is significantly faster at only a few seconds if speed of analysis is of greater importance than filtering for lower quality sequences. The tight clustering of motif ratios within different genes, coupled with the large number of sequence reads associated with PCR products, indicates that low levels of index hopping [55] is unlikely to pose any issues if the workflow is performed on patterned flow cell instruments. We found that low levels of cross contamination (for example, from clipping regimes or sample processing) does not cause a significant issue in genotyping, and could be filtered out in the analysis if the WT:mutant ratio is outside of normal limits for the gene in question.

### 4.1 Use of NGS for genotyping mutations in gene families

By harnessing the parallel nature of NGS, non-specific or closely related sequences can be easily filtered out, enhancing the accuracy of the genotyping result, especially for mutations in gene families. Sanger sequencing of the wild type allele of Sirpb1a, for example, differed from the other family members by fewer than 10 bp. As initial QC is performed on heterozygotes, slight mismatches in the primer sequence may result in amplification of the wild type amplicon being too strong for the analyst to confidently separate true from background signal. Certainly, from our own experience in attempting to genotype in this manner, the analysis is complicated and potentially error prone, especially when no exogenous sequences such as selection markers are present. The use of NGS and placement of unique motifs for filtering (Supplemental Data S6) removes this issue, giving a much higher confidence in the results. We therefore highly recommend when processing exon deletions with high homology, especially those with minimal mismatches within the assay, that alternative assays and sequencing are performed as an additional QC step during heterozygote × heterozygote cohort matings. This will ensure that potential homozygotes are not mis-genotyped, and the strain mistakenly assigned as homozygous-lethal. Non-Mendelian genotype ratios can also offer a vital clue that the assays are not performing as expected (for example, all heterozygote offspring from a het × het mating), especially later in the breeding scheme.

## CRediT authorship contribution statement

**Diane Gleeson:** Investigation, Methodology, Writing - review & editing. **Debarati Sethi:** Investigation. **Radka Platte:** Investigation. **Jonathan Burvill:** Investigation. **Daniel Barrett:** Investigation. **Shaheen Akhtar:** Investigation. **Michaela Bruntraeger:** Resources. **Joanna Bottomley:** Project administration. **Sanger Mouse Genetics Project:** . **James Bussell:** Conceptualization, Supervision. **Edward Ryder:** Conceptualization, Investigation, Software, Supervision, Visualization, Writing - original draft, Writing - review & editing.

## Declaration of Competing Interest

The authors declare that they have no known competing financial interests or personal relationships that could have appeared to influence the work reported in this paper.
