# Supplementary material for: High-throughput genotyping of high-homology mutant mouse strains by next-generation sequencing
Source: Methods. 2021 Jul;191:78–86. doi: 10.1016/j.ymeth.2020.10.011 (PMC8205115; doi:10.1016/j.ymeth.2020.10.011)
Supplement: Supplementary data 1 [file mmc1.docx]

**Supplementary information**

**S1. End point PCR reagents and cycling conditions**

| **Reagent** | **volume (µl)** |
| --- | --- |
| Gene specific Primer 1 (10µM) | 0.3 |
| Gene specific Primer 2 (10µM) | 0.3 |
| Gene specific Primer 3 (10µM) | 0.3 |
| MgCl2 (50mM) | 0.45 |
| 10x Buffer | 1.5 |
| dNTP (100mM) | 0.15 |
| Platinum Taq (Invitrogen) | 0.15 |
| ddH_2_O | 10.85 |
| DNA | 1 |
| **Total** | **15** |

PCR cycling conditions

| 1    94 ^o^C    5 min |
| --- |
| 2    94 ^o^C    30 sec |
| 3   58 ^o^C    30 sec |
| 4    72 ^o^C    45 sec |
| 5    Go to ‘2’ + 34 cycles |
| 6    72 ^o^C    5 min |
| 7    12 ^o^C    forever |

**S2. qPCR reagents and cycling conditions**

| **Reagent** | **Volume (µl)** |
| --- | --- |
| 2x GTXpress buffer | 5 |
| 20x TaqMan assay | 0.5 |
| ddH_2_O | 3 |
| 20x endogenous probe (Tfrc) | 0.5 |
| DNA | 1 |

qPCR cycling conditions

| 1    95 ^o^C    20 sec |
| --- |
| 2    95 ^o^C    10 sec |
| 3   60 ^o^C    30 sec |
| 4    72 ^o^C    45 sec |
| 5    Go to ‘2’ + 34 cycles |

**S3. Overall ratios of NGS genotypes.**


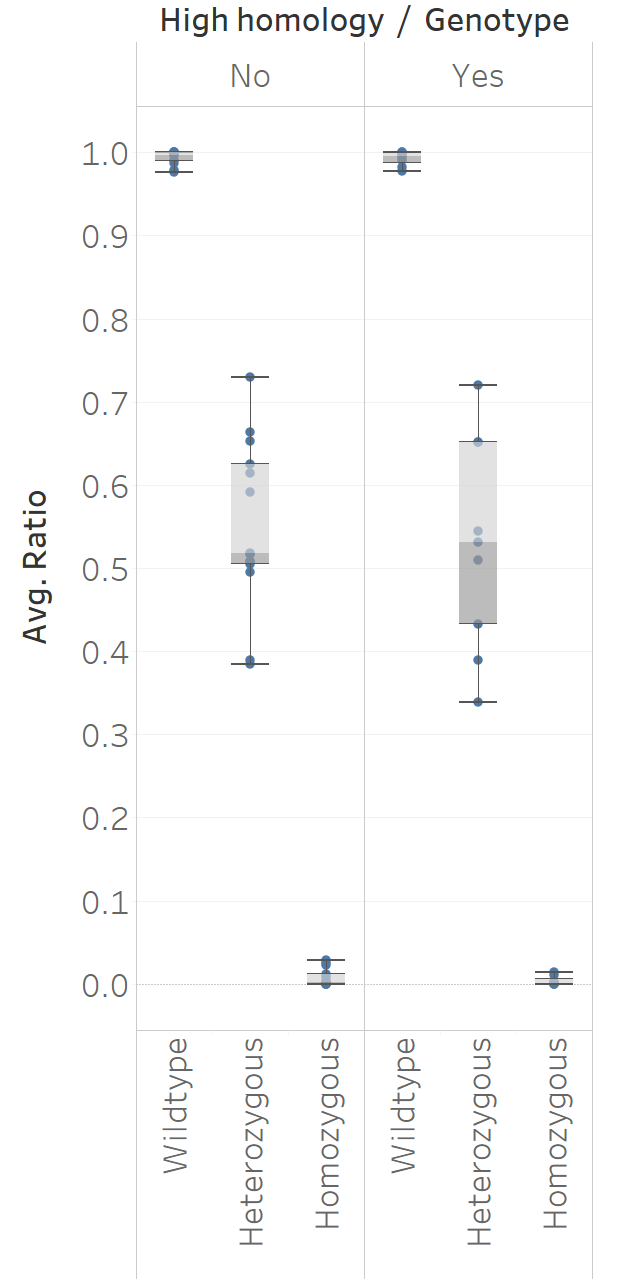


**S4. Homology within Psg target regions.**

Psg21 has a 97% sequence match to Psg23 across the entire deletion and flanking areas (2.4Kb), and numerous matches to other Psg genes (eg Psg27) throughout the region. This makes designing qPCR assays extremely challenging.

Psg23 CATAATCACCCCTTTGTACCAAAAAAATAATTGAAGATACTCTGGAAGATTGTGAACACG

Psg21 CATAATCACCCCTTTGTACCAAAAAAATAATTGAAGATACTCTGGAAGATTGTGAACACG

Psg27 CATAATCACCCCTTTGTACCAAAAAAATAATTGAAGATACTCTGGAAGATTGTGAACACG

************************************************************

Psg23 GAGAAGAACGCTTCCTCCTTCAGCAACGCTGGGCGGCACTGATTCAATAGTGAGCTTGGC

Psg21 GAGAAGAACGCGTCCTCCTTCAGCAACTCTGGGCGGCACTGATTCAATAGTGAGCTTGGC

Psg27 GAGAAAAACACTTCCTCCTTCAGCAACTCTGGGCGGCACTGATTCAATAGTGAGCTTGGC

***** *** * *************** ********************************

Psg23 AGGGAAGGAAGGACGCCCACAGGTGAAATGAGACGCTAGAAGGGAAGAGAGATCCACTGA

Psg21 AGGGAAGGAAGGACGCCCACAGGTGAAATGAGAGGCTAGAAGGGAAGAGAGATCCATCGA

Psg27 AGGGAAGGAAGGGCGCCCACAGGTGAAATGAGAGGCTAGAAGGGAAGAGAGATCCATCGA

************ ******************** ********************** **

Psg23 TATCAGGACTTTCATATTTGACTGAGATGGCACCCTGTGTCCTCATCAGCTGTGTCCATA

Psg21 TATTAGGACTTTCATATTTGACTGAGATGGCACCCTGTGTCCTGATCAGCTGTGTCCATA

Psg27 TATTAGGACTTTCATATTTGACTGAGATGGCACCCTGTGTCCTGATCAGCTGTGTCCATA

*** *************************************** ****************

**S5. Spiked cross-contamination experiment results.**


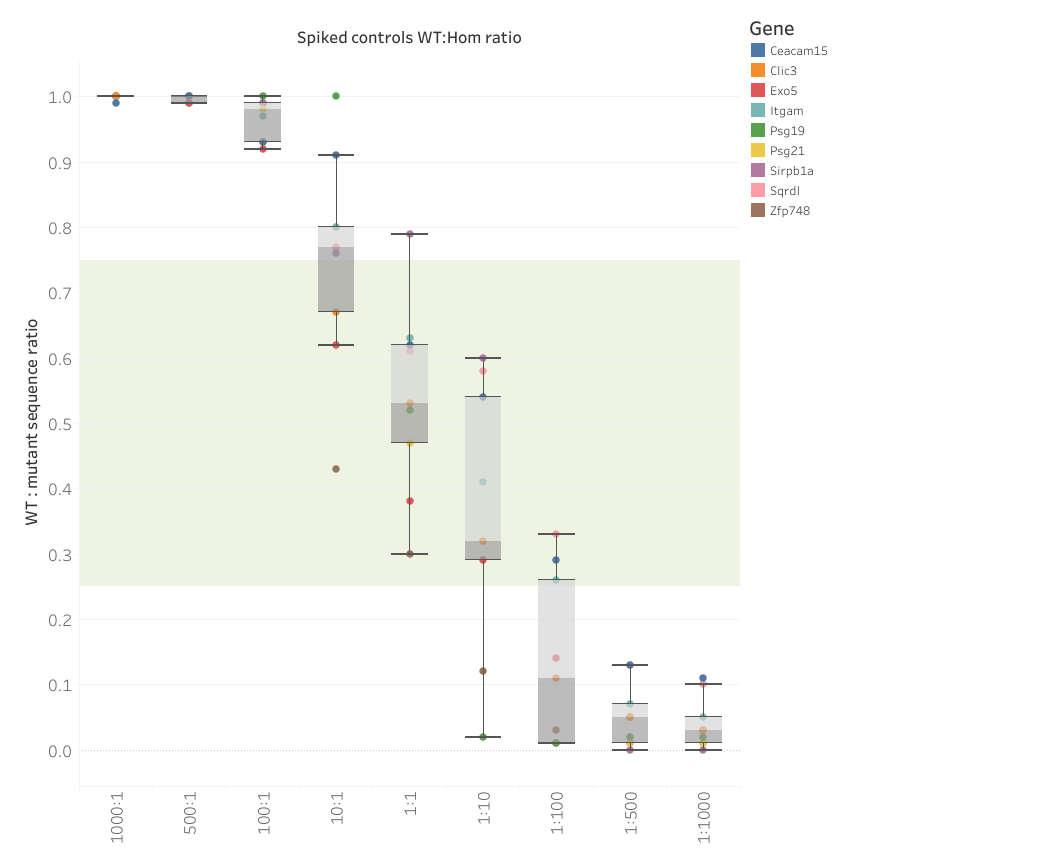


**S6. Filtering motifs for Sirpb1a-c.**

The Sirpb1a-c genes are highly homologous across the 5’ boundary of the exon deletion, making designing a discriminatory assay particularly challenging. By placing the genotyping sequencing motifs at distinct locations, the correct gene can be assigned.

Sirpb1a ATTCCAGTTCAGGCAGTGCTCAAAATTCTGGCTCTATTATAAGAGACAATGTTCTTACCT 60

Sirpb1c ATTCCAGTTCAGGCAGTGCTCAAAATTCTGGCTCTATTATGTGAGACAATATTCTTACCT 60

Sirpb1b ATTCCAGTTCAGG**CAGTGCTCAGAATTCTGGCTCTATTATATGAGA**-------------- 46

********************** ***************** ****

Sirpb1a CTCCTGTGACAGTTCAGGTAATTAACTGGAATCCTTTACT**TTCAATGGTAAGAGTAGCTC** 120

Sirpb1c CTCCTGTGACAGTTCAGATAATTAACTGGAATCCTTTACTTTCAATGGTAAGAGTAGC**TC** 120

Sirpb1b -**CAATGTGACAGTTCAGG**TAATTAACTGGAATCCTTTGCTTTCAATGGTAAGGGTAGCTC 105

************* ******************* ************** *******

Sirpb1a **TGAGACAGGGAGATGATACTATTGTTGGGG**CATGCTCACCTTTAAATCCCAGAAGCAGGA 180

Sirpb1c **TGAAGCAGGGAGATGATTCTATTGTTGGGGCATGCTCACCTTTAAGTC**CCAGAAGCAGGA 180

Sirpb1b TGAGGCAGGGAGATTATTCTGTTTTTGGGGCATGCTCACCTTTAAGTCCCAGAAGCAGGA 165

*** ********* ** ** ** ********************* **************

Sirpb1a TCAACAGCAGGACACTGTGAGGAATGTGGGTCCAGGCATCTAGGAGAAGCATGGTTGGTG 240

Sirpb1c TCAACAGCAGGACACAGTGAGGAATGTGGGTCCAGGCATCTAGGAGAAGCATGGTTGGTG 240

Sirpb1b TCAACAGCAGGACACTATGAGGAATGTGGGTCCAGGCATCTAGGAGAAGCATGGTTGGTG 225

*************** *******************************************

ACAGCA deleted region

**AATGGT** Wild type sequence filter tag

**S7. Quality control and genotyping workflow of CRISPR-mediated exon deletions**

**
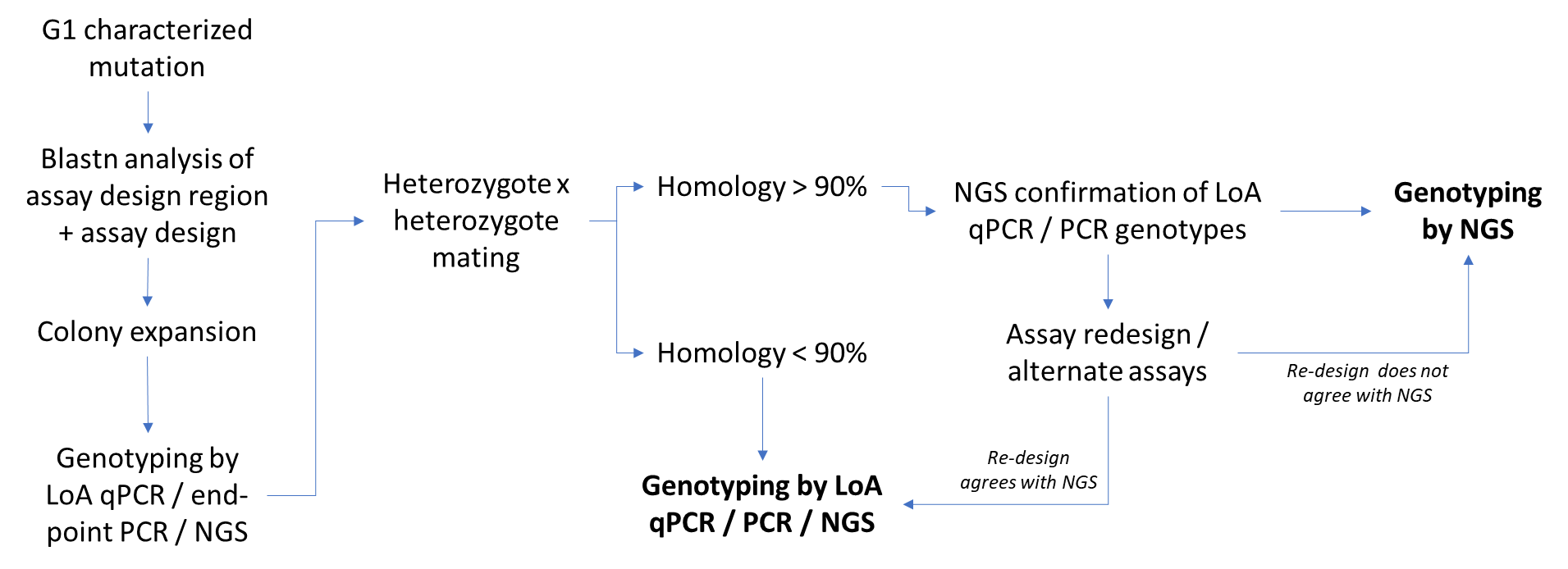
**
